# Absolute and Relative Morbidity Burdens Attributable to Various Illnesses and Injuries Among Active Component Members of the U.S. Armed Forces, 2023

**Published:** 2024-06-20

**Authors:** Armed Forces Health Surveillance Division Defense Health Agency, US

## Abstract

**What are the new findings?:**

In 2023, injuries, mental health disorders, and musculoskeletal diseases were the medical conditions associated with the most medical encounters, greatest numbers of service members affected, and highest numbers of hospital days. Major category conditions increased overall by about 17% compared to 2022, and medical encounters increased by 18%. COVID-19 accounted for no more than 0.3% of total member medical encounters and hospital bed days among active component service members in 2023.

**What is the impact on readiness and force health protection?:**

The major condition categories in this report present health challenges among U.S. active component service members that can affect force readiness. Investigating morbidity and health care burdens enables prioritization of relevant health conditions, and their primary causes, for appropriate resource allocation and proactive management of potential health events through timely intervention, research, and resources. Development and consistent implementation of policies and safeguards is critical for reducing the burden of diseases that affect readiness.

## 1 BACKGROUND

*MSMR*’s annual burden of disease reports are designed to provide accurate estimations of the general health status of U.S. military personnel, for prioritization of effective interventions with measurable impacts on force readiness.^1^ In these reports, diagnoses are grouped to inform our readership of the major factors and variables each year affecting health care provision within the Military Health System (MHS). Although burden of disease within a health care system can be classified into several categories, the majority of the disease burden globally comes from non-communicable diseases (NCDs), with communicable diseases the second-most prevalent, followed by maternal, neonatal and nutritional diseases, and subsequently injuries.^2^

To broadly describe the morbidity burden among active component service members (ACSMs), *MSMR* has used, since 2001, a classification system derived from the Global Burden of Disease (GBD) Study,^3,4^ a systematic, scientific effort that began 30 years ago to quantify the magnitude of all major diseases, their risk factors, and intermediate clinical outcomes in a highly standardized manner. This systematic classification enables comparisons between populations and health problems over time.^5^ *MSMR* utilizes the GBD classification system in combination with an International Classification of Diseases, 10th Revision, Clinical Modification (ICD-10-CM) chapter-based system for categorization of hospitalizations and ambulatory visits among the MHS population.

To improve the usefulness of this information, these classification schemes are refined by *MSMR*’s editorial staff. The major classification system for diagnoses, ICD-10-CM, features over 68,000 separate codes.^5^ While the ICD-10-CM is organized in logical chapters, the groupings are not optimal for describing burdens of disease in a military population. Consequently, some re-groupings of diagnoses are necessary to achieve a meaningful portrayal of the burden in the military population.

The burden of disease in a young, healthy, predominantly male service member population will differ substantially from that of the general U.S. or global populations. Service applicants are medically screened prior to military service to ensure fitness requirements for physically demanding jobs, and throughout their service mandatory periodic (typically annual) health assessments and screenings among ACSMs may detect conditions potentially undetected in other populations. The numerous readiness-related outpatient visits required for each ACSM, the prescribed circumstances of military living, training requirements, and access to medical care without cost may contribute to different morbidity burden profiles compared to other population groups.

Individuals enlist or are commissioned into the active component typically between the ages of 17 and 25, with almost all members ending service by age 50. In 2022, the mean age of ACSMs in the U.S. Armed Forces was approximately 29, with 1.3% of the population over 50.^3^ By contrast, the median age of the U.S. population in 2022 was 38.9, with 36.1% over age 50.^4^ Women constituted 19.2% of the active component in 2023, compared to 51.0% in the general U.S. population.^6^

Within the military population and its specific environment, categories of illnesses and injuries requiring hospitalization have historically differed from those that result in the most outpatient visits. The added requirements for readiness are likely a major factor in outpatient health care provision, but rarely for hospitalization. The categories of medical conditions and readiness requirements that account for the most medical encounters overall may differ from those that affect the most individuals or have the most debilitating or long-lasting effects.^4^

This annual summary uses several health care burden measures to quantify the impacts in 2023 of various illnesses and injuries among members of the active component of the U.S. Armed Forces. Health care burden metrics include the total number of medical encounters, individuals affected, and hospital bed days. A consistent and comparative description of the burden of diseases and injuries, and subpopulations affected, is an important input to health decision-making and planning processes and can provide valuable information for where changes in policy or preventive emphasis may improve the medical readiness of the force.^7^

## 2 METHODS

The population for this analysis includes all individuals who served in the active component of the Army, Navy, Air Force, Marine Corps, or Space Force at any time during the surveillance period of January 1, 2023 through December 31, 2023. Each service member contributed encounters and person-time only for the actual months served during the surveillance period. All data in this analysis were derived from records maintained in the Defense Medical Surveillance System (DMSS), which documents both ambulatory care encounters and hospitalizations of active component members of the U.S. Armed Forces. DMSS contains all encounters in military medical and civilian treatment facilities when reimbursed through the MHS. Encounters not routinely and completely documented within fixed military and non-military hospitals and medical clinics (e.g., during deployments, field training exercises, or at sea) are excluded from this analysis.

DMSS data for all inpatient and outpatient medical encounters of ACSMs during the surveillance period were summarized according to the primary (first-listed) diagnosis if reported with an ICD-10 code between A00 and T88, an ICD-10 code beginning with Z37 (outcome of delivery), or Department of Defense (DOD) unique personal history codes DOD0101–DOD0105 (personal history of traumatic brain injury). All illness- and injury-specific diagnoses, as defined by ICD-10 codes, were grouped into 25 burden of disease-related ‘categories’ and 153 ‘conditions’, which are described as major category conditions in this report, based on a modified version of the classification system developed for the GBD Study.^4^ This classification system was developed by the *MSMR* editorial staff in 2001 and is updated annually.

The GBD system groups diagnoses with common pathophysiologic or etiologic bases or significant DOD health policy importance. In this article, some diagnoses grouped into single categories in the GBD system (e.g., mental health disorders) were disaggregated to increase military relevance. In addition, injuries are classified by affected anatomic site rather than cause, as external causes of injuries using NATO Standardization Agreement (STANAG) 2050 codes are incompletely reported in military outpatient records.^8^

The morbidity burdens attributable to various conditions were estimated based on the total number of medical encounters associated with each condition, i.e., total hospitalizations and ambulatory visits for the condition with a limit of 1 encounter for an individual per condition each day; and numbers of service members affected by each condition, i.e., individuals with at least 1 medical encounter for the condition during the year; as well as total bed days during hospitalizations for each condition.

## 3 RESULTS

**Morbidity Burden, by Category**

Provisional data indicate that affected ACSMs (n=584,756) experienced medical encounters due to injury more than any other morbidity-related category in 2023 (**Figure 1a**). Ranking third in terms of hospital bed days, this major burden of disease category accounted for about one-fourth (23.2%) of all medical encounters (**Figure 1b**). The injury category combines ICD-10 S (injury) and T codes (burns and poisonings); however, injuries account for nearly 98% of ambulatory encounters within the category (data not shown).

Mental health disorders accounted for more hospital bed days (n=213,905) than any other morbidity-related category, contributing over half (54.8%) of all hospital bed days, ranking fifth for individuals affected (**Figures 1a** and **1b**). Together, injury and mental health disorders accounted for over two-thirds (64.8%) of all hospital bed days and 42.3% of all medical encounters.

Maternal conditions (e.g., pregnancy complications and delivery) accounted for a relatively large proportion of all hospital bed days (n=56,122; 14.4%) but a much smaller proportion of medical encounters overall (n=205,381; 1.5%) (**Figures 1a** and **1b**). As women comprised only 19.2% of the active duty force in 2023, these summary statistics understate the impact of these conditions among that group. Maternal conditions were the most frequent category among women in the active component.

**Medical Encounters, by Condition**

**Figure 2:**
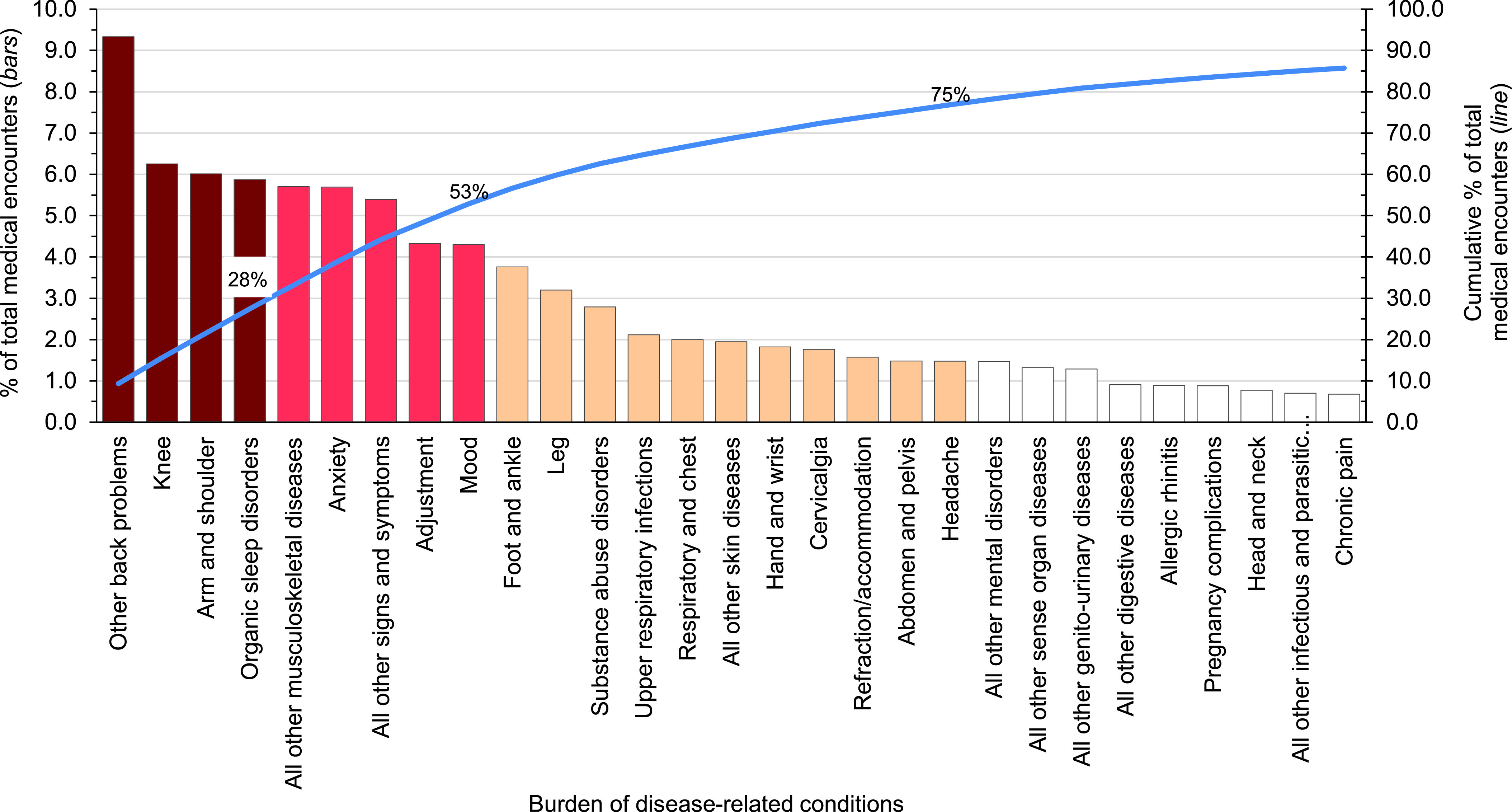
Percentage and Cumulative Percentage Distribution, Burden of Disease-related Conditions that Accounted for the Most Medical Encounters, Active Component, U.S. Armed Forces, 2023

In 2023, 5 burden of disease-related conditions accounted for almost one-third (33.2%) of all illness- and injury-related medical encounters: other back problems (e.g., lower back pain, other dorsalgia), organic sleep disorders (e.g., insomnia, obstructive sleep apnea), all other signs and symptoms (e.g., fever, headache, general signs and symptoms not otherwise specified), knee injuries, and arm/shoulder injuries (**Figure 2**). Moreover, the 10 conditions associated with the most medical encounters constituted more than half (56.6%) of all illness- and injury-related medical encounters.

The health conditions that accounted for the most medical encounters among ACSMs in 2023 were predominantly injuries, mental health disorders, and musculoskeletal diseases. Of reported injuries, knee (6.3%), arm/shoulder (6.0%), foot/ankle (3.8%), and leg (3.2%) resulted in the most medical encounters (**Figure 2** and **Table**). Mental health disorder diagnoses resulted most frequently from anxiety (5.7%), adjustment (4.3%), mood (4.3%), and substance abuse disorders (2.8%). Other back problems (9.3%), all other musculoskeletal diseases (5.7%; e.g., pain in foot, pain in leg), and cervicalgia (1.8%) generated the most medical encounters from musculoskeletal diseases. COVID-19 accounted for 0.3% of total medical encounters, ranking 44th in 2023, continuing the decrease to 1.4% of total encounters seen in 2022.

**Individuals Affected, by Condition**

In 2023, the 10 conditions that affected the most service members were signs, symptoms, and other ill-defined conditions (all other signs and symptoms and respiratory/chest); musculoskeletal diseases (other back problems and all other musculoskeletal diseases); respiratory infections (upper respiratory infections); neurological conditions (organic sleep disorders); respiratory and chest, sense organ diseases (refraction/accommodation); injuries (knee and arm/shoulder); and skin diseases (all other skin diseases). COVID-19 affected 32,508 service members and ranked thirty-fifth for numbers affected, a considerable decrease in rank from twelfth in 2022.

**Hospital Bed Days, by Condition**

**Figure 3:**
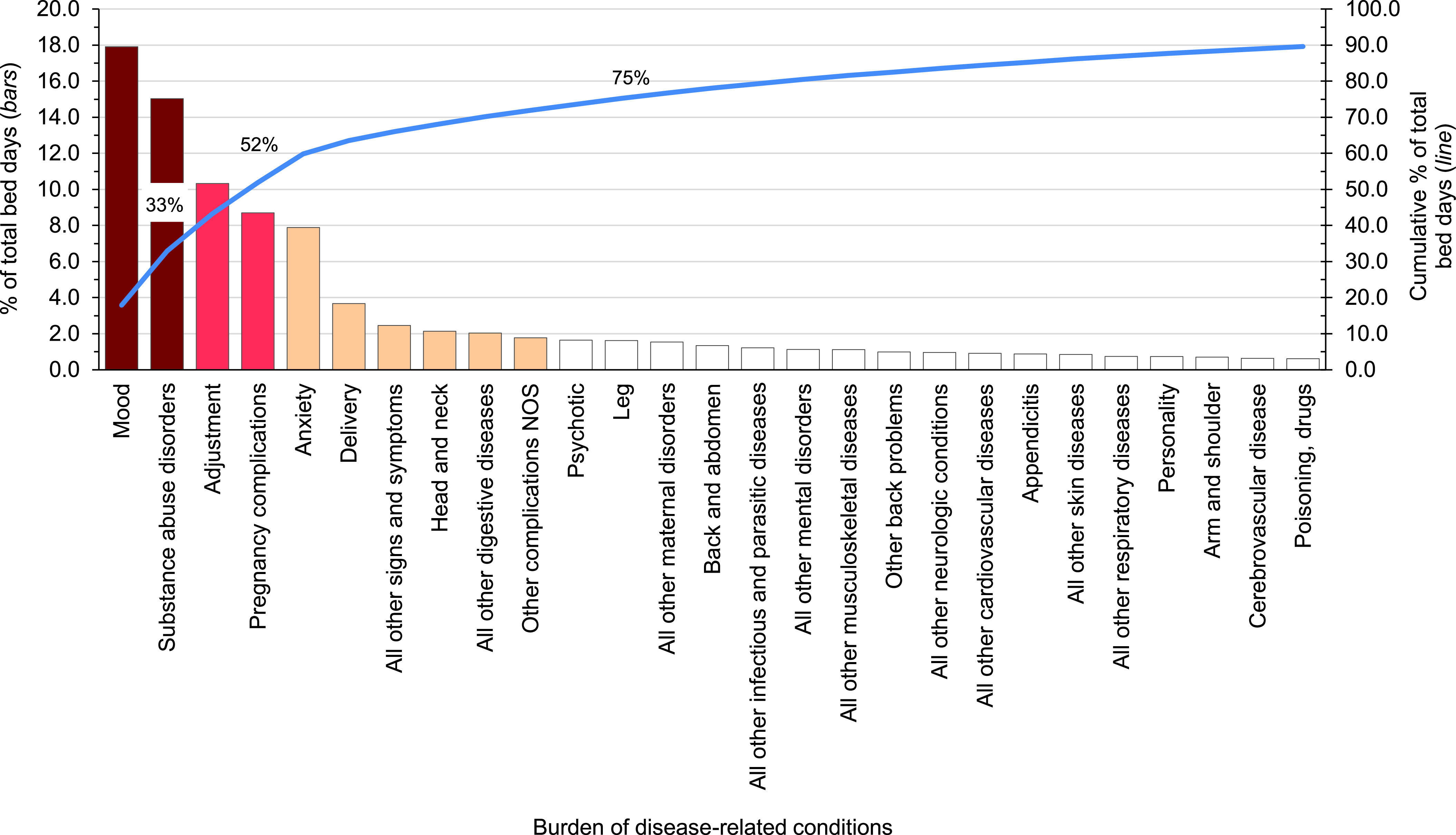
Percentage and Cumulative Percentage Distribution, Burden of Disease-related Conditions that Accounted for the Most Hospital Bed Days, Active Component, U.S. Armed Forces, 2023

Mood and substance abuse disorders accounted for nearly one-third (32.9%) of all hospital bed days (**Figure 3**) in 2023. Four mental health disorders (mood, substance abuse, adjustment, anxiety) and 2 maternal conditions (pregnancy complications, delivery) together accounted for almost two-thirds (63.5%) of all hospital bed days (**Table** and **Figure 3**). About 10% of all hospital bed days were attributable to injuries and poisonings. COVID-19 accounted for 0.11% of total hospital bed days among ACSMs, down from 0.3% in 2022 (**Table**).

**Relationships Between Health Care Burden Indicators**

There was a strong positive correlation between numbers of medical encounters attributable to various conditions and numbers of individuals affected by those conditions (*r*=0.87) (data not shown). The 3 leading causes of medical encounters were among the 5 conditions that affected the most individuals (**Table**), while weak-to-moderate positive relationships were detected for hospital bed days attributable to conditions and numbers of individuals affected (*r*=0.22) by, or medical encounters associated (*r*=0.41) with, those conditions. For example, substance abuse disorders and labor and delivery were among the top-ranking conditions, by proportion of total bed days, but these conditions affected relatively few ACSMs in 2023.

## 4 DISCUSSION

This *MSMR* report provides the most recent data available for a major disease matrix comparable to previous reports. Compared to 2022, overall major category conditions reported in 2023 increased by 16.9%, medical encounters increased by 18%, as well as individuals affected (8.6%) and hospital bed days (5.7%). This result is consistent with the major findings of prior *MSMR* reports on morbidity and health care burdens among U.S. military members.

Injuries, mental health disorders, and musculoskeletal disorders were the medical conditions in 2023 associated with the most medical encounters, highest numbers of affected service members, and greatest numbers of hospital days. Only 9 of the 153 burden of disease conditions comprising this report, or 5.8% of the listed conditions, accounted for slightly more than half of all illness- and injury-related medical encounters: 2 anatomic site-defined injuries (knee and arm/shoulder), 3 mental health disorders (anxiety, adjustment, and mood disorders), organic sleep disorders, 2 musculoskeletal conditions (other back problems and all other musculoskeletal diseases), and all other signs and symptoms. Injuries were the single leading cause of death, disability, hospitalization, outpatient visits, and manpower loss among U.S. military service members in 2023.^9^

The pattern of illness and injury among U.S. active component members is distinct from other population groups with different demographic distributions and occupational hazards, such as the general U.S. population and non-service member MHS beneficiaries. Injuries, mental health disorders, and musculoskeletal conditions are identified in the literature as the leading causes of morbidity and disability among service members throughout military history, affecting readiness and health care provision.^9,10,11^

Due to lifestyles that can be influenced by operational conditions, multiple combat missions, separation from family, among other factors, a number of mental disorders including occupational stress, depression, and suicide are common among military personnel.^10^ Some studies have reported significant associations between major depressive disorder and deployment.^11^ Exposure to intense physical demands in training and operational environments increases risk of musculoskeletal injury, which contributes to significant morbidity among military personnel.^12^ With psychosocial factors shown to be implicated in increased risk of developing back pain, approaching this and related issues holistically, rather than divided among discrete categories, would be beneficial.^13,14^ Holistic, integrated approaches to care that not only reflect the identified burden of conditions and associated risk factors, foremost the unique health challenges that result from the unique complexities of service experience and the nature of combat, but which also consider the interplay between military and civilian health care systems would better meet the health needs of military personnel and veterans.^15^

Because an understanding of the associations between preventive health care and disease occurrence is required for prevention of injury and disease among service members, a comprehensive medical surveillance system is necessary for routine injury and disease monitoring and data-informed prioritization of research and successful prevention programs. These surveillance, analysis, and reporting efforts can culminate in effective partnerships between commanders, policy-makers, and service members for direct actions to prevent disease and injuries.^8,11^ Reporting on the burden of disease and injury includes reliable quantification of their physical and psychosocial health impacts, as well as risk factors, that can provide valuable information about the health status of a population, allowing optimal resource allocation for prevention and treatment. An accurate estimate of the health status of the armed forces can be used not only for determining expected health care use and costs and the prioritization of effective interventions, but evaluations of their impacts and cost-effectiveness.^16^ Recent and accurate information on the scale of health disorders among service members, groups noticeably at risk, and trends in their health statuses over time are critical data for policy-makers.

## Figures and Tables

**Figure 1a:**
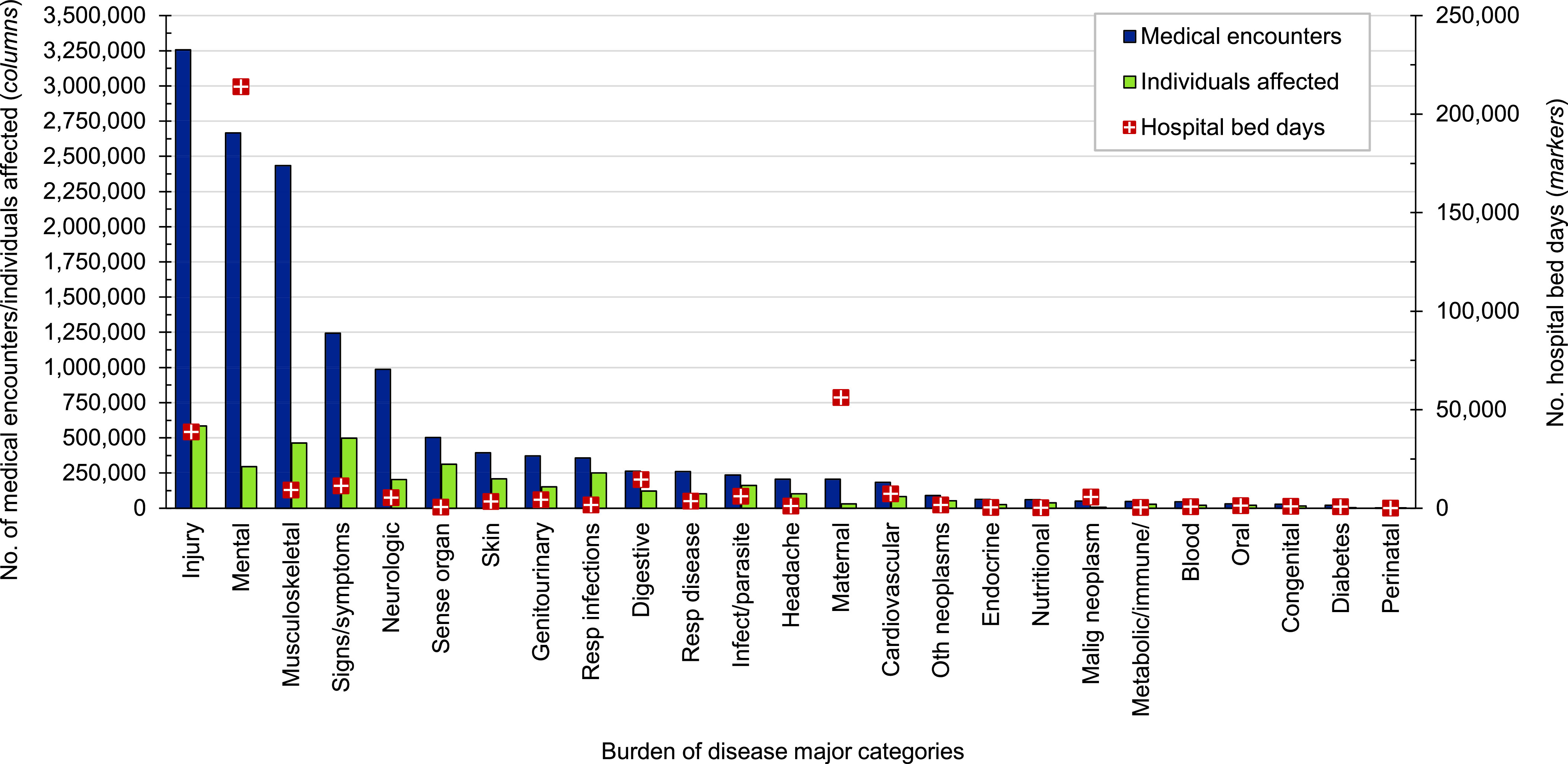
Numbers of Medical Encounters, Individuals Affected, and Hospital Bed Days by Burden of Disease Category, Active Component, U.S. Armed Forces, 2023

**Figure 1b:**
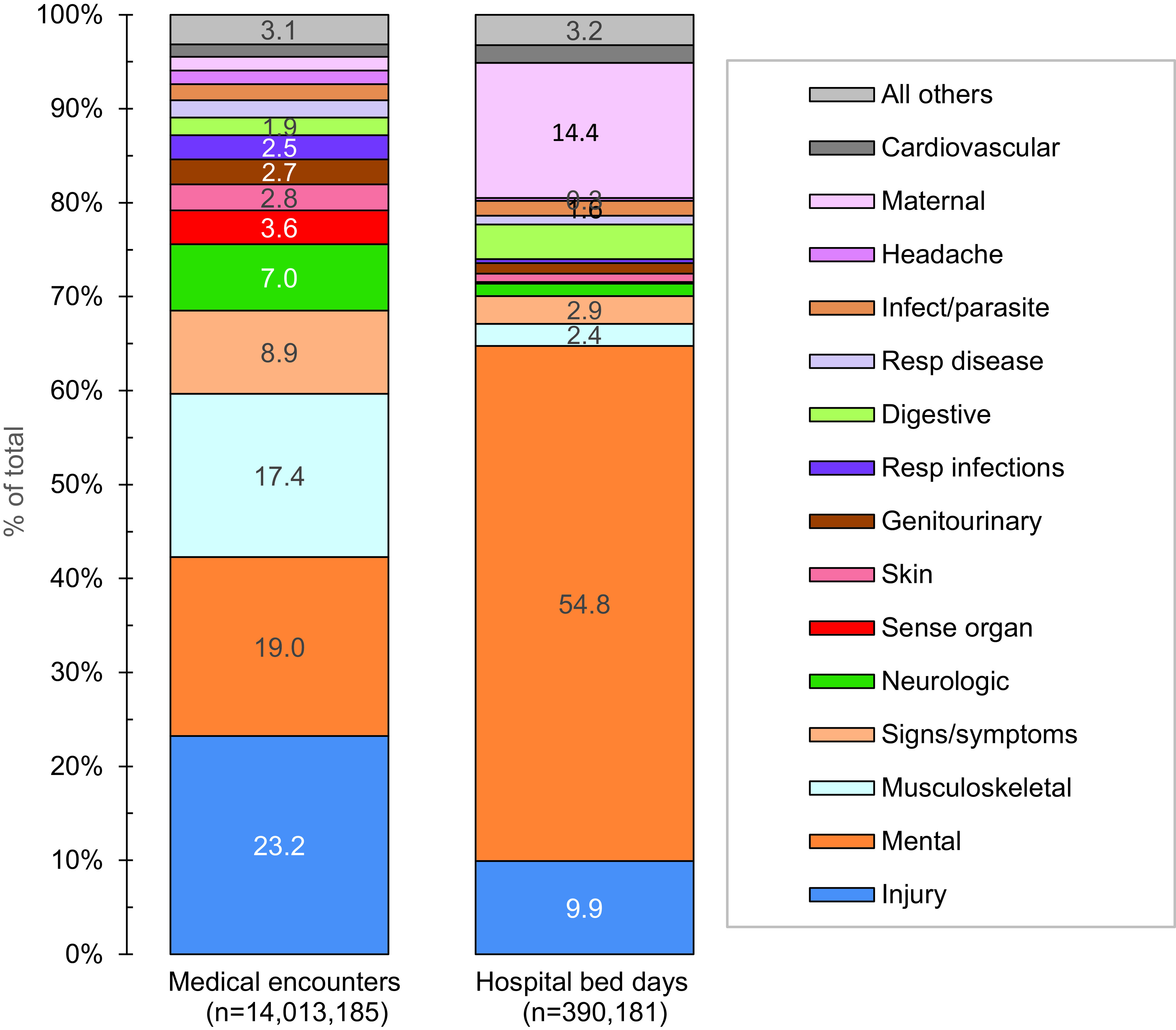
Percentage of Medical Encounters and Hospital Bed Days Attributable to Burden of Disease Major Categories, Active Component, U.S. Armed Forces, 2023

**Table:** Health Care Burdens Attributable to Various Diseases and Injuries, Active Component, U.S. Armed Forces, 2023

Major Category Condition^a^	Medical Encounters^b^		Individuals Affected^c^		Hospital Bed Days	
	No.	Rank^d^	No.	Rank^d^	No.	Rank^d^
Total	14,013,183				390,131	
**Injury and poisoning**	**3,256,538**				**38,804**	
Knee	876,325	2	174,885	6	1,315	33
Arm and shoulder	842,568	3	161,996	9	2,750	25
Foot and ankle	527,007	10	151,575	11	1,887	28
Leg	448,323	11	109,812	16	6,338	12
Hand and wrist	255,273	16	90,252	18	1,059	41
Head and neck	108,422	27	55,380	25	8,341	8
Back and abdomen	60,202	34	32,366	36	5,243	14
Other complications NOS	34,301	47	18,717	56	6,933	10
Unspecified injury	33,459	49	23,083	43	1,013	42
Other harm from external causes	29,058	51	18,231	57	467	58
Environmental	23,218	63	17,255	58	678	49
Poisoning, non-drug	5,663	104	4,300	92	269	73
Poisoning, drugs	4,406	109	1,670	107	2,407	27
All other injury	3,404	115	2,874	101	84	95
Other superficial injury	2,875	119	895	116	0	146
Other burns	1,982	122	1,260	111	20	125
Under-dosing	52	153	35	149	0	146
**Mental health disorders**	**2,667,599**				**213,905**	
Anxiety	797,775	6	131,309	12	30,773	5
Adjustment	606,459	8	123,984	14	40,298	3
Mood	603,069	9	81,931	20	69,881	1
Substance abuse disorders	391,221	12	32,883	33	58,642	2
All other mental disorders	206,544	21	65,027	23	4,410	16
Personality	23,337	61	4,473	91	2,881	24
Psychotic	18,197	70	1,790	106	6,415	11
Somatoform	11,765	83	3,674	95	598	50
Tobacco dependence	9,232	92	5,153	86	7	135
**Musculoskeletal diseases**	**2,435,474**				**9,200**	
Other back problems	1,307,669	1	262,406	2	3,857	18
All other musculoskeletal diseases	799,349	5	256,586	3	4,365	17
Cervicalgia	247,305	17	64,733	24	65	100
Osteoarthritis	45,513	41	21,300	47	482	57
Other knee disorders	16,875	72	7,351	71	393	66
Other shoulder disorders	14,711	76	6,666	76	21	123
Rheumatoid arthritis	4,052	112	1,412	109	17	128
**Signs, symptoms and ill-defined conditions**	**1,243,239**				**11,408**	
All other signs and symptoms	755,414	7	342,543	1	9,596	7
Respiratory and chest	280,180	14	167,654	8	571	54
Abdomen and pelvis	207,645	19	128,778	13	1,241	36
**Neurologic conditions**	**987,668**				**5,307**	
Organic sleep disorders	822,662	4	171,501	7	467	58
Chronic pain	95,075	29	29,887	38	151	84
All other neurologic conditions	48,314	40	19,045	54	3,753	19
Other mononeuritis, upper and lower limbs	13,129	80	6,587	77	81	97
Epilepsy	5,650	105	1,833	105	704	48
Multiple sclerosis	2,528	120	534	124	145	86
Parkinson's disease	310	141	54	143	6	138
**Sense organ diseases**	**502,266**				**653**	
Refraction/accommodation	220,800	18	181,554	5	2	144
All other sense organ diseases	185,094	22	117,145	15	580	53
Hearing disorders	83,058	31	50,643	26	56	106
Glaucoma	11,534	84	7,022	72	7	135
Cataracts	1,780	123	945	114	8	133
**Skin diseases**	**394,675**				**3,404**	
All other skin diseases	273,044	15	154,704	10	3,333	22
Sebaceous gland diseases	72,647	33	41,140	29	19	127
Contact dermatitis	48,984	38	36,404	31	52	107
**Infectious and parasitic diseases**	**235,726**				**6,075**	
All other infectious and parasitic diseases	98,261	28	66,029	22	4,761	15
COVID-19	37,674	44	32,508	35	415	63
Unspecified viral infection	26,843	54	24,886	41	36	114
Tinea skin infections	26,659	55	21,096	48	1	145
Diarrheal diseases	18,825	69	16,245	59	549	55
STDs	17,200	71	12,401	66	99	92
Chlamydia	7,897	95	6,798	75	12	132
Hepatitis B and C	1,374	127	601	122	4	139
Tuberculosis	377	139	144	137	126	89
Intestinal nematode infection	291	142	257	132	0	146
Malaria	163	148	81	140	52	107
Bacterial meningitis	84	150	22	151	20	125
Tropical cluster	78	151	54	143	0	146
**Respiratory infections**	**357,149**				**1,626**	
Upper respiratory infections	296,346	13	220,640	4	404	64
Lower respiratory infections	31,935	50	21,892	45	1,158	39
Otitis media	28,868	52	22,984	44	64	102
**Respiratory diseases**	**261,491**				**3,635**	
Allergic rhinitis	124,460	25	43,220	27	14	129
All other respiratory diseases	56,981	35	33,671	32	2,899	23
Asthma	34,333	46	15,298	60	293	71
Chronic sinusitis	22,110	64	14,884	61	61	103
Deviated nasal septum	15,885	73	8,731	69	254	75
Chronic obstructive pulmonary disease	7,722	97	6,522	78	114	90
**Genitourinary diseases**	**371,675**				**4,357**	
All other genito-urinary diseases	180,463	23	87,746	19	1,543	30
Female genital pain	56,729	36	24,471	42	49	109
Menstrual disorders	34,265	48	21,392	46	353	67
UTI and cystitis	25,531	58	19,535	52	151	84
Other breast disorders	24,753	60	13,136	65	432	61
Vaginitis and vulvitis	19,158	68	14,336	63	0	146
Kidney stones	15,780	74	6,873	74	537	56
Nephritis and nephrosis	11,520	85	4,824	89	1,259	35
Benign prostatic hypertrophy	3,476	113	2,169	103	33	115
**Digestive diseases**	**262,723**				**14,504**	
All other digestive diseases	127,115	24	66,707	21	7,959	9
Esophagus disease	52,789	37	31,545	37	595	51
Other gastroenteritis and colitis	43,319	43	27,298	39	1,671	29
Constipation	20,215	67	14,247	64	80	98
Inguinal hernia	10,211	89	4,098	94	212	77
Appendicitis	6,842	100	2,895	100	3,443	21
Peptic ulcer disease	1,427	125	910	115	223	76
Cirrhosis of the liver	805	132	172	135	321	69
**Maternal conditions**	**205,381**				**56,122**	
Pregnancy complications	123,573	26	25,828	40	33,955	4
All other maternal disorders	44,052	42	12,086	67	6,008	13
Delivery	21,524	65	9,582	68	14,338	6
Ectopic/miscarriage/abortion	10,117	90	4,598	90	431	62
Puerperium complications	6,115	102	3,359	97	1,390	31
**Headache**	**206,991**				**1,189**	
Headache	206,991	20	102,239	17	1,189	38
**Cardiovascular diseases**	**184,895**				**7,364**	
All other cardiovascular diseases	87,003	30	42,158	28	3,570	20
Essential hypertension	76,957	32	40,894	30	163	82
Cerebrovascular disease	9,546	91	2,354	102	2,483	26
Ischemic heart disease	7,760	96	3,053	99	834	44
Inflammatory	2,973	118	1,430	108	307	70
Rheumatic heart disease	656	135	558	123	7	135
**Other neoplasms**	**90,871**				**1,696**	
All other neoplasms	48,481	39	32,640	34	1,229	37
Benign skin neoplasm	25,219	59	19,884	51	3	141
Lipoma	10,434	88	6,359	80	26	119
Uterine leiomyoma	6,737	101	3,141	98	438	60
**Endocrine disorders**	**62,747**				**492**	
Hypothyroidism	15,457	75	7,647	70	39	112
Other thyroid disorders	14,205	77	5,610	84	266	74
Testicular hypofunction	13,705	78	5,510	85	0	146
All other endocrine disorders	13,327	79	6,876	73	179	80
Polycystic ovarian syndrome	6,053	103	3,452	96	8	133
**Malignant neoplasms**	**50,535**				**5,675**	
All other malignant neoplasms	7,707	98	1,220	112	1,268	34
Lymphoma and multiple myeloma	7,648	99	655	121	723	47
Breast cancer	5,216	106	463	129	134	88
Melanoma and other skin cancers	5,208	107	2,165	104	59	104
Leukemia	4,686	108	336	131	770	46
Testicular cancer	4,332	110	664	119	107	91
Colon and rectum cancers	4,282	111	352	130	882	43
Brain	3,435	114	232	133	1,123	40
Thyroid	2,208	121	475	128	175	81
Prostate cancer	1,402	126	221	134	31	116
Mouth and oropharynx cancers	1,083	128	133	138	83	96
Cervix uteri cancer	1,030	129	524	125	27	118
Stomach cancer	601	136	51	145	91	93
Trachea, bronchus, and lung cancers	555	137	51	145	38	113
Liver cancer	290	143	39	148	40	111
Pancreas cancer	244	144	35	149	26	119
Ovary cancer	203	146	44	147	21	123
Bladder cancer	197	147	57	142	4	139
Esophagus cancer	132	149	9	153	14	129
Corpus uteri cancer	76	152	19	152	59	104
**Metabolic and immunity disorders**	**47,638**				**488**	
Lipoid metabolism disorders	25,584	57	18,865	55	65	100
Other metabolic disorders	11,079	87	5,711	83	328	68
Gout	7,974	94	4,150	93	23	122
Immunity disorders	3,001	116	955	113	72	99
**Nutritional disorders**	**61,588**				**265**	
Overweight, obesity	35,561	45	21,039	49	41	110
All other nutritional disorders	25,818	56	19,486	53	137	87
Protein, energy malnutrition	209	145	81	140	87	94
**Blood disorders**	**45,740**				**886**	
Other non-deficiency anemias	12,865	81	6,475	79	287	72
All other blood disorders	12,520	82	6,093	81	400	65
Iron-deficiency anemia	11,372	86	4,897	88	155	83
Hereditary anemias	8,165	93	5,796	82	31	116
Other deficiency anemias	818	131	499	127	13	131
**Oral conditions**	**30,189**				**1,327**	
All other oral conditions	28,510	53	20,561	50	1,324	32
Dental caries	924	130	880	117	0	146
Periodontal disease	755	134	701	118	3	141
**Congenital anomalies**	**27,974**				**977**	
All other congenital anomalies	23,258	62	14,464	62	592	52
Congenital heart disease	2,974	117	1,262	110	198	78
Other circulatory anomalies	1,742	124	657	120	187	79
**Diabetes mellitus**	**20,816**				**744**	
Diabetes mellitus	20,816	66	5,025	87	794	45
**Conditions arising during the perinatal period^e^**	**1,595**				**28**	
All other perinatal anomalies	762	133	507	126	25	121
Low birth weight	497	138	157	136	3	141
Birth asphyxia and birth trauma	336	140	129	139	0	146

Abbreviations: No., number; NOS, not otherwise specified; UTI, urinary tract infection; STDs, sexually transmitted diseases.

^a^ Burden of disease major categories and burden of disease-related conditions based on a modified version of those defined in the Global Burden of Disease Study.^3,4^

^b^ Medical encounters include total hospitalizations and ambulatory visits for the condition (with no more than 1 encounter per individual per day per condition).

^c^ Individuals with at least 1 hospitalization or ambulatory visit for the condition.

^d^ Rank is based on the number of encounters, individuals affected, or hospital bed days in respective columns within the listing of the 153 burden-related disease conditions. For individuals affected, 4 pairs of tied values (n=81; n=54; n=51; n=35) were given the same ranking (140; 143; 145; 149). For hospital bed days, there were 8 conditions with the rank of 146 (0); 26 other conditions had tied rankings.

^e^ Conditions affecting newborns erroneously coded on service member medical records.
